# Recurrent Fibro-Osseous Pseudotumour of the Digit Mimicking Infection: A Narrative Review and Case Report

**DOI:** 10.1177/15589447261481209

**Published:** 2026-09-04

**Authors:** Elijah J. C. Wong, Brahman Sivakumar, Luke McCarron, Craig Buchan, David J. Graham

**Affiliations:** 1 Griffith University, Southport, QLD, Australia; 2 Royal North Shore Hospital, St Leonards, NSW, Australia; 3 Hornsby Ku-ring-gai Hospital, NSW, Australia; 4 Nepean Hospital, Kingswood, NSW, Australia; 5 The University of Sydney, Camperdown, NSW, Australia; 6 Australian Research Collaboration on Hands (ARCH), Mudgeeraba, QLD, Australia; 7 Bond University, Robina, QLD, Australia; 8 Gold Coast Hospital and Health Service, Southport, QLD, Australia; 9 South Coast Radiology, Mermaid Beach, QLD, Australia; 10 The University of Queensland, Herston, Australia; 11 Queensland Children’s Hospital, South Brisbane, Australia

**Keywords:** fibro-osseous pseudotumour, digit, FOPD, recurrent lesion, finger infection, histopathology

## Abstract

**Background:**

Fibro-osseous pseudotumour of the digit (FOPD) is a rare, benign soft-tissue lesion that mimics infection and malignancy due to its rapid growth, pain, and radiological features. Misdiagnosis can result in inappropriate treatment, including unnecessary ablative surgery.

**Methods:**

A narrative literature review was conducted using PubMed and Embase, supplemented by reference screening. Case reports and case series of FOPD involving the digits of the hands or feet were included. Data were synthesised to identify clinical, histopathological, radiological, and management patterns. In addition, we report a recurrent case of FOPD misdiagnosed as tenosynovitis.

**Results:**

Twenty-two case reports published between 2015 and 2025 and four large case series prior to 2015 yielded a total of 134 patients. Fibro-osseous pseudotumor of the digit typically affects young to middle-aged adults (mean age, 36 years) with a slight female predominance. Lesions presented as rapidly enlarging painful nodules, occasionally associated with prior trauma, and were frequently mistaken for infection or extraskeletal osteosarcoma. Complete surgical excision was the definitive treatment, with recurrence reported rarely and almost exclusively following incomplete excision. Our case demonstrated recurrence after initial debridement, with subsequent definitive excision leading to functional recovery, although residual stiffness and sensory disturbance persisted.

**Conclusions:**

Fibro-osseous pseudotumor of the digit should be considered in the differential diagnosis of rapidly enlarging digital lesions. Complete surgical excision is curative in most cases, while molecular markers such as ubiquitin-specific peptidase 6 may refine the diagnosis in morphologically ambiguous presentations. Future research should prioritise the development of standardised diagnostic criteria, prognostic markers, and long-term outcome reporting.

## Introduction

A fibro-osseous pseudotumour of the digit (FOPD) is a rare, benign soft-tissue lesion characterised by abnormal ossification occurring near the digits, particularly in the hands but also occurring in the foot.^1,2^ First described in 1986, FOPD occurs adjacent to, but does not arise from, bone and is therefore considered a soft-tissue lesion.^1^ Within the literature, it has been referred to by various names such as “florid reactive periostitis,” “parosteal fasciitis,” and “fasciitis ossificans.”^1,2^

Clinically, FOPD most commonly affects young female adults aged between 20 and 40 years, although cases have been reported from as young as 5 years to as old as 81 years.^2-4^ These lesions typically present as localised fusiform swellings of the digit with pain and erythema.^1,2,5,6^ While some patients develop functional limitation or ulceration, others remain asymptomatic.^3^ Diagnosis is usually confirmed by characteristic histopathological and radiological findings; however, because of their rapid onset and growth, FOPD is frequently mistaken for malignancy or infection.^3,5^

Management involves complete surgical excision and is usually curative.^1-3,5^ The prognosis is excellent with good functional recovery following surgery. However, delayed or incorrect diagnosis, particularly when FOPD is misinterpreted as osteosarcoma, may result in unnecessary ablative surgery, including amputation, or inappropriate surgical debridement and antibiotic therapy if treated as an infection.^1,3,5,6^

Despite these important clinical implications, the literature describing the clinical, histopathological, and radiological features of FOPD remains limited. Given the rarity of these lesions and the predominance of single-case reports, this narrative review and case report aim to synthesise existing knowledge and identify recurring diagnostic patterns.

## Methods

A narrative literature review was conducted using the PubMed and Embase databases. Given the absence of a comprehensive review within the last decade and the rarity of FOPD, studies published from 2015 onwards were prioritised. Earlier evidence was also incorporated, primarily from 4 large case series (Dupree and Enzinger,^1^ de Silva and Reid,^5^ Moosavi et al,^6^ and Chaudhry et al^7^), to ensure coverage of key historical data and to capture thematic developments in clinical presentation, diagnostic practices, and management.

The search strategy employed the terms “fibro-osseous pseudotumour,” “fibro-osseous pseudotumor,” “FOPD,” “digit*,” and “hand*,” “foot*” with appropriate Boolean operators to enhance specificity to digital lesions. Reference lists of included articles were screened for additional studies. Studies were eligible for inclusion if they were published in English and reported clinical outcomes in patients with FOPD of the digits of the hands or feet. Titles and abstracts were screened for relevance, followed by full-text review.

Given the rarity of FOPD, all included studies were descriptive in nature, consisting of individual case reports and small series. These were synthesised to identify recurring clinical, histopathological, and radiological features, as well as management strategies and outcomes.

The accompanying case report was prepared according to the CAse REport (CARE) guidelines.^8^ Informed written consent was obtained from the patient. Clinical information, diagnostic workup, operative details, and follow-up data were extracted from electronic medical records spanning presentation to 4 months post the operation. All data were de-identified and cross-checked for accuracy and completeness by clinical specialists.

## Results

The literature search identified 22 case reports published between 2015 and 2025. When combined with 4 larger case series (Dupree and Enzinger,^1^ de Silva and Reid,^5^ Moosavi et al,^6^ and Chaudhry et al^7^), a total of 134 cases of FOPD were included in this review. A total of 108 cases of FOPD (80.6%) were located in the upper extremity, and 25 cases were found in the feet or toes (18.7%). One lesion was located on the forehead. Study characteristics are summarised in Table 1.

**Table 1.** Study Characteristics of FOPD Case Reports.

Author	Country of study	Study characteristics (sample size, sex, age, anatomical site)	Presenting symptoms	Investigations	Treatment	Follow-up
Chaudhry et al^7^	United Kingdom	n = 17 Female, n = 9 Male, n = 8 Median age = 34 years Hand/finger, n = 8 Foot/toe, n = 8 Forehead, n = 1	Painful, enlarging nodules (digits), swelling, erythematous; rapid onset weeks–months	Histopathology (fibroblasts, osteoid, giant cells, atypia mild), radiology not always reported	Excision	No recurrence (6-24 months follow-up)
Cho et al^9^	South Korea	n = 1 27-year-old male Foot/toe	Enlarging erythematous subungual nodule, painful swelling	Histopathology (fibroblasts, osteoid), MRI (T1 low, T2 high)	Excision	No recurrence at 6 months
Choi et al^10^	South Korea	n = 1 68-year-old male Hand/finger	Painful enlarging nodule mimicking sarcoma	Histopathology, immunohistochemistry (calponin, SMA positive; S100, desmin negative)	Excision	No recurrence (6 months)
Dupree and Enzinger^1^	United States	n = 21 Female, n = 13 Male, n = 8 Median age = 33 years Hand/finger, n = 20 Foot/toe, n = 1	Painful enlarging lesions, swelling, erythema	Histopathology classic features (fibroblasts, osteoid, no zoning)	Excision	No recurrence documented
Flucke et al^11^	Netherlands	n = 5 Female, n = 3 Male, n = 2 Mean age = 48 years Hand/finger, n = 4 Foot/toe, n = 1	Painful swelling, enlarging nodules	Histopathology, USP6 FISH testing	Excision	No recurrence (short-term follow-up)
Gómez-Zubiaur^12^	Spain	n = 1 13-year-old female Foot/toe	Digit mass mimicking pyogenic granuloma, ulcerated	Histopathology, radiology not detailed	Excision	No recurrence
Grover et al^4^	India	n = 1 35-year-old male Foot/toe	Painful swelling, enlarging nodule	Histopathology, radiology not detailed	Excision	No recurrence
Isaac et al^13^	United States	n = 1 24-year-old female Foot/toe	Painful recurrent nail bed mass	Histopathology confirmed recurrence	Excision (repeat)	Recurrence once, no recurrence after re-excision
Jawadi et al^14^	Saudi Arabia	n = 1 27-year-old female Hand/finger	Rapidly enlarging painful swelling encircling neurovascular bundle	MRI, histopathology	Excision	No recurrence
Li et al^15^	China	n = 1 25-year-old male Hand/finger	Painful rapidly enlarging digit lesion	Histopathology	Excision	Recurrence once
Liu et al^16^	China	n = 1 32-year-old male Hand/finger	Digit mass, swelling, painful	Histopathology	Excision	No recurrence
Meani et al^17^	Australia	n = 1 16-year-old male Foot/toe	Subungual painful swelling	Histopathology, radiology	Excision	No recurrence
Moosavi et al^6^	United States	n = 43 Female, n = 26 Male, n = 17 Median age = 40 years Hand/finger, n = 42 Foot/toe, n = 1	Painful, enlarging masses, erythematous, ulcerated in some	Histopathology classic features	Excision	No recurrence (majority)
De Silva and Reid^5^	United Kingdom	n = 14 Female, n = 7 Male, n = 7 Mean age = 38.1 years Hand/finger, n = 14	Rapidly growing masses, painful	Histopathology	Excision	No recurrence
Nunes Pombo et al^18^	Portugal	n = 1 68-year-old female Hand/finger	Ulcerated mass	Histopathology	Excision	No recurrence
Putnam et al^19^	United States	n = 1 26-year-old female Foot/toe	Painful lower extremity mass (rare site)	Histopathology	Excision	No recurrence
Rela and Bantick^20^	United Kingdom	n = 1 60-year-old female Hand/finger	Painful mass, diagnostic challenge	Histopathology	Excision	No recurrence
Sakuda et al^21^	Japan	n = 1 30-year-old male Hand/finger	Rapidly enlarging painful lesion	MRI, histopathology	Excision	No recurrence
Singal et al^22^	India	n = 1 36-year-old female Foot/toe	Subungual mass, painful swelling	Histopathology	Excision	No recurrence
Singh et al^23^	United Kingdom	n = 1 6-year-old female Hand/finger	Digit swelling, painful	Histopathology	Excision	No recurrence
Švajdler et al^24^	Czech Republic	n = 12 Female, n = 6 Male, n = 6 Median age = 32 years Hand/finger, n = 8 Foot/toe, n = 4	Painful enlarging nodules	Histopathology, molecular (COL1A1-USP6)	Excision	No recurrence
Takahashi et al^25^	Japan	n = 1 36-year-old male Foot/toe	Painful enlarging digit lesion	Histopathology	Excision	No recurrence
Tuzzato et al^26^	Italy	n = 1 22-year-old female Hand/finger	Painful hand swelling	Histopathology	Excision	No recurrence
Wang et al^27^	China	n = 2 Female, n = 1 Male, n = 1 Mean age = 25 years Hand/finger, n = 2	Painful digit lesions	Histopathology, molecular USP6 fusions	Excision	No recurrence
Zhang et al^28^	China	n = 2 Male, n = 2 Mean age = 42 years Foot/toe, n = 2	Painful enlarging nodules	Histopathology, molecular USP6 fusions	Excision	No recurrence
Zhou et al^3^	Australia	n = 1 48-year-old male Hand/finger	Painful swelling digit	Histopathology	Excision	No recurrence

Abbreviations: COL1A1, collagen type I alpha 1 chain; FISH, fluorescence in situ hybridisation; FOPD, fibro-osseous pseudotumour of the digit; MRI, magnetic resonance imaging; n, number of patients; S100, S100 protein; SMA, smooth muscle actin; USP6, ubiquitin-specific peptidase 6.

A slight majority of the cohort was female (74/134; 55%), with a mean age of 36 years (range, 6-68). Most cases occurred in young to middle-aged adults. The United States, the United Kingdom, and China contributed nearly half of all studies (n = 13).

Most cases developed spontaneously without preceding trauma; however, 8 studies described a history of trauma or repetitive overuse preceding symptom onset.^1,4,5,7,9,13,17,28^ Fibro-osseous pseudotumour of the digit typically presented as a rapidly enlarging nodular lesion, with growth over weeks to months,^3,6,7,9,10,13-17,19-23,25,26^ although 1 report described a 2-year progression.^4^ Accompanying features included swelling, erythema, and pain, with ulceration less frequently reported. Lesions were most often confined to the dermis or subcutaneous tissue, without continuity to bone. Rarely, the lesion extended around the neurovascular bundle or produced cortical erosion.^9,12,14,15,21,22,26^

Histology consistently demonstrated fibroblastic proliferation with mild cytological atypia and spindle cell morphology.^3,5-7,9,10,14-18,20,23-26,28^ Osteoid formation was a hallmark finding, frequently occurring at the periphery of the lesion.^3-7,9-11,13-15,17,18,20-26^ Osseous trabeculae were typically arranged irregularly without a peripheral zoning pattern, helping to differentiate FOPD from myositis ossificans.^1,3,4,6,7,9,12,13,24,27^ Multinucleated giant cells were variably present, while periosteal reaction was occasionally described.^1,4-6,14-17^ Limited immunohistochemistry showed negativity for cytokeratin, S100, and desmin, with positivity for smooth muscle actin (SMA) and calponin, supporting a myofibroblastic phenotype.^7,10,13^ More recent reports incorporated molecular testing, with ubiquitin-specific peptidase 6 (USP6) gene rearrangements detected in 11 of 14 tested cases across 4 studies.^11,24,27,28^

Imaging typically revealed a well-circumscribed soft-tissue mass, often separated from the underlying bone by a radiolucent cleft. Magnetic resonance imaging (MRI) findings included low signal intensity on T1 and high signal intensity on T2 sequences,^14-16,21,22,26^ while 1 ultrasound report described a hyperechoic lesion with posterior acoustic shadowing.^4^ Cortical erosion was an uncommon finding, reported in a small number of cases.^9,12^

Treatment was almost universally surgical, with complete local excision as the standard of care.^1,4-7,9,10,13-23,26,28^ Amputation was rarely performed and only performed in the setting of extensive involvement or misdiagnosis.^1,7^ Recurrence was infrequent and primarily occurred following incomplete excision.^1,5-7,13,18,19,21^ Follow-up periods ranged from 6 to 24 months, with no recurrence reported in most cases.^1,3,4,6,7,9,10,13,17-23,26^ However, long-term functional outcomes were inconsistently described across studies, limiting assessment of patient recovery.

A recurring theme across the literature was initial misdiagnosis, with FOPD commonly mistaken for infection,^1,5-7,14^ tenosynovitis,^1,5-7^ or malignancy, particularly extraskeletal osteosarcoma.^1,3,5-7,9,10,14-27^ This highlights the risk of unnecessary aggressive intervention if FOPD is not correctly identified.

## Case Report

A 46-year-old man presented to the emergency department of a secondary hospital in Southeast Queensland with a 3-week history of painful swelling at the dorsal aspect of the distal interphalangeal (DIP) joint in the left index finger. He recalled no recent antecedent trauma, although he reported a minor crush injury to the fingertip several years prior. He denied systemic symptoms, as well as relevant medical and family history.

Prior to hospital presentation, the patient had consulted his general practitioner and was treated with oral steroids and empiric antibiotics for 3 days without improvement, on the suspicion of infected gout. Three separate ultrasound scans suggested possible tenosynovitis, though inflammatory markers and routine blood tests were unremarkable. The patient also underwent 2 debridements, which yielded scant blood and no purulent fluid.

**Figure 1.**
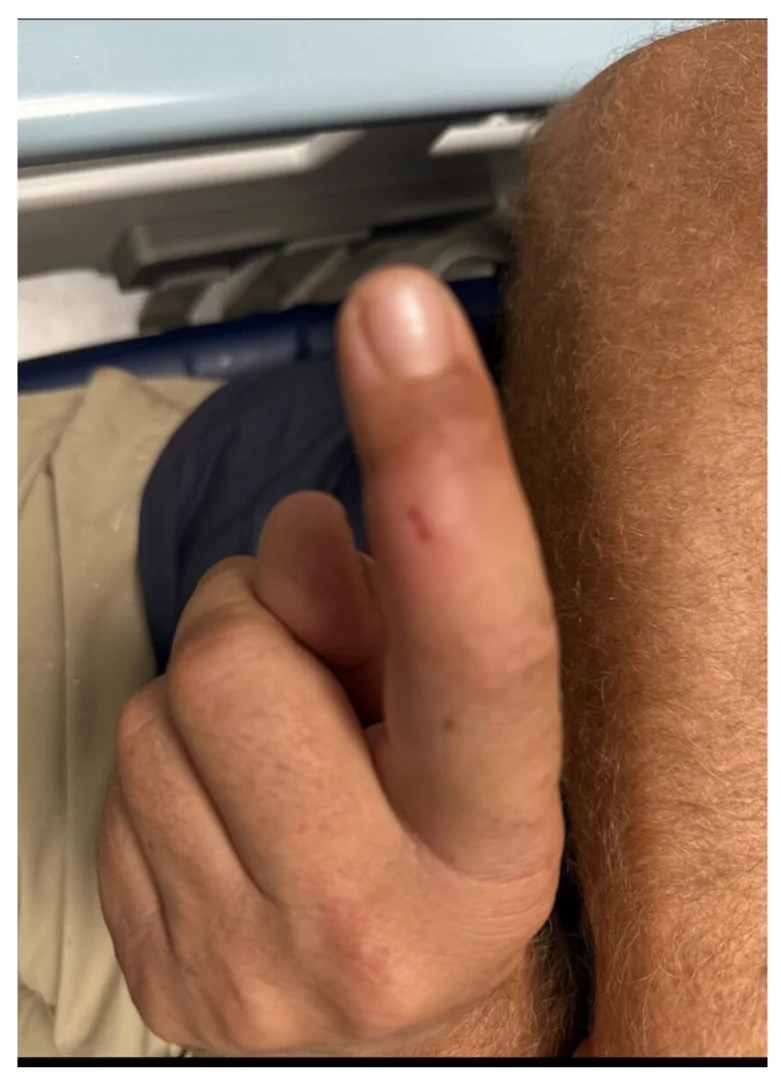
Photo of pre-operative lesion on left index finger.

**Figure 2.**
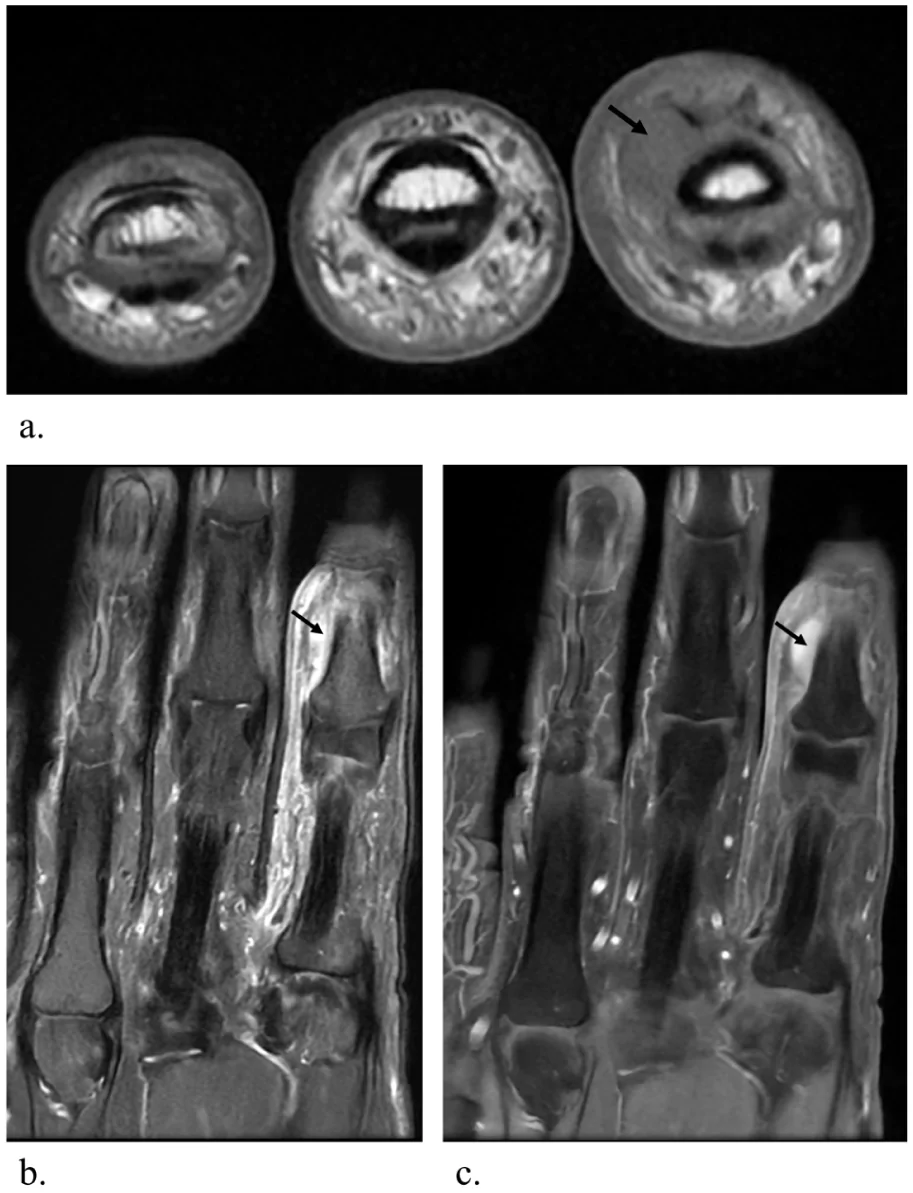
Initial presentation of the left hand on magnetic resonance imaging. (A) Axial T1: Deep subcutaneous/parosteal mass (black arrow) on the dorsal/ulnar border of the second middle phalanx, which demonstrates ill-defined low signal with infiltration of the conjoint lateral bands of the extensor mechanism. Note the absence of a T1 high signal intensity wall (penumbra sign). (B) Coronal T2 FS: The mass (black arrow) demonstrates intermediate to high signal intensity with no significant reactive marrow oedema. (C) Coronal T1 FS post-gadolinium: Uniform enhancement of the mass (black arrow) post-gadolinium administration. No medullary cavity involvement. NB: Radiographs were not initially obtained, and T2*/diffusion-weighted imaging sequences were not performed. FS indicates fat suppression.

On hospital presentation, the DIP joint appeared fusiform, mildly erythematous, and tender, but the digit was neurologically intact (Figure 1). An MRI of the left index finger demonstrated a deep dorsal-ulnar parosteal soft-tissue mass adjacent to the middle phalanx (Figure 2). Plain radiographs were not initially obtained. The patient was booked for a same-day admission and underwent surgical debridement with a flexor sheath washout. Intraoperatively, turbid fluid was observed within the flexor sheath, and a 20 x 10 x 5-mm biopsy specimen was sent for microbiology and histopathology.

Post-operatively, he was discharged on oral amoxicillin and clavulanic acid and referred for hand therapy. Cultures were negative; however, histology demonstrated atypical osteoblastic proliferation with irregular immature bone trabeculae and surrounding fibroblastic proliferation, raising concern for a neoplastic process.

**Figure 3.**
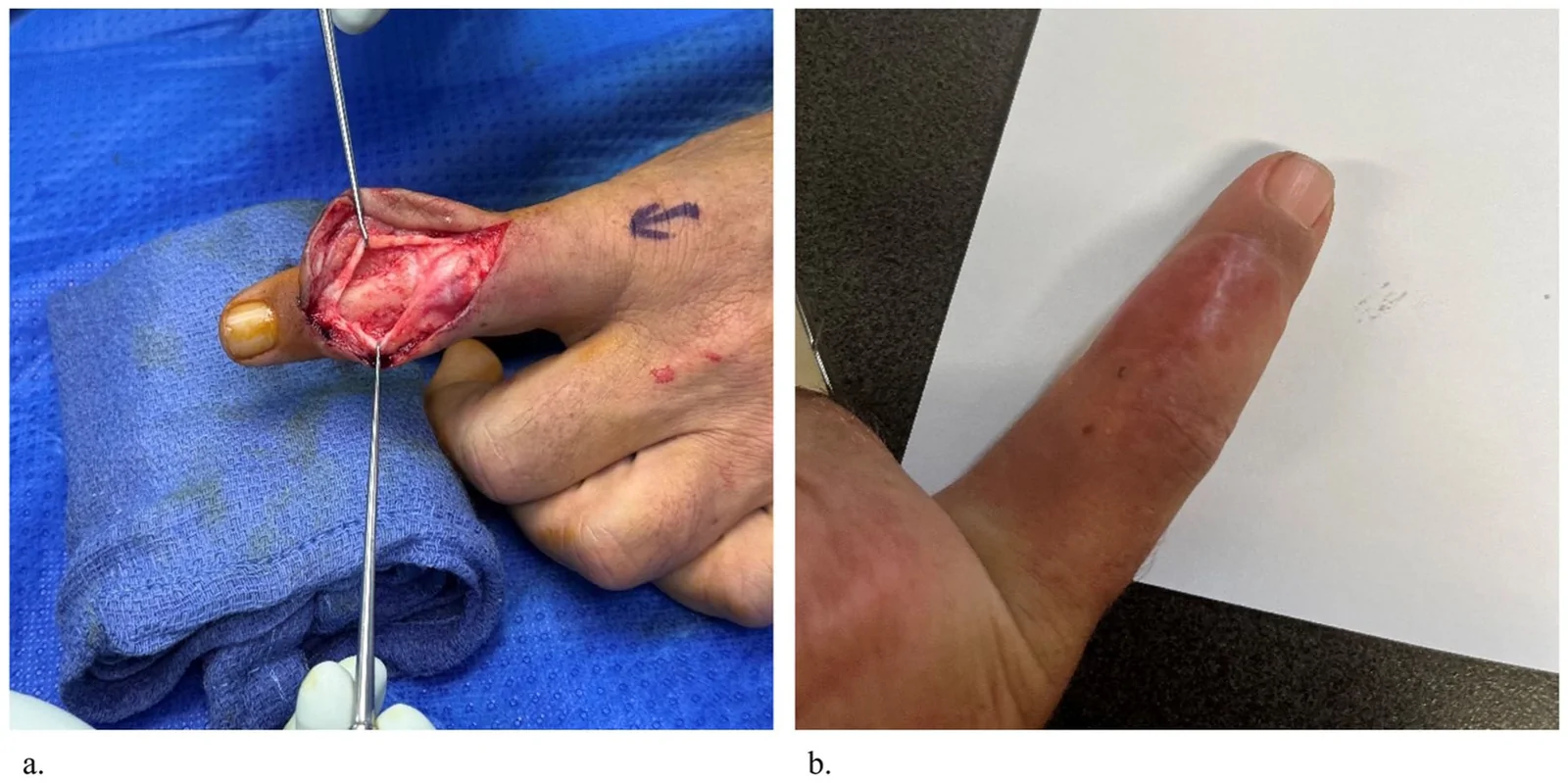
(A) Surgical debridement with washed flexor sheath. (B) Post-operative photo of the left index finger.

**Figure 4.**
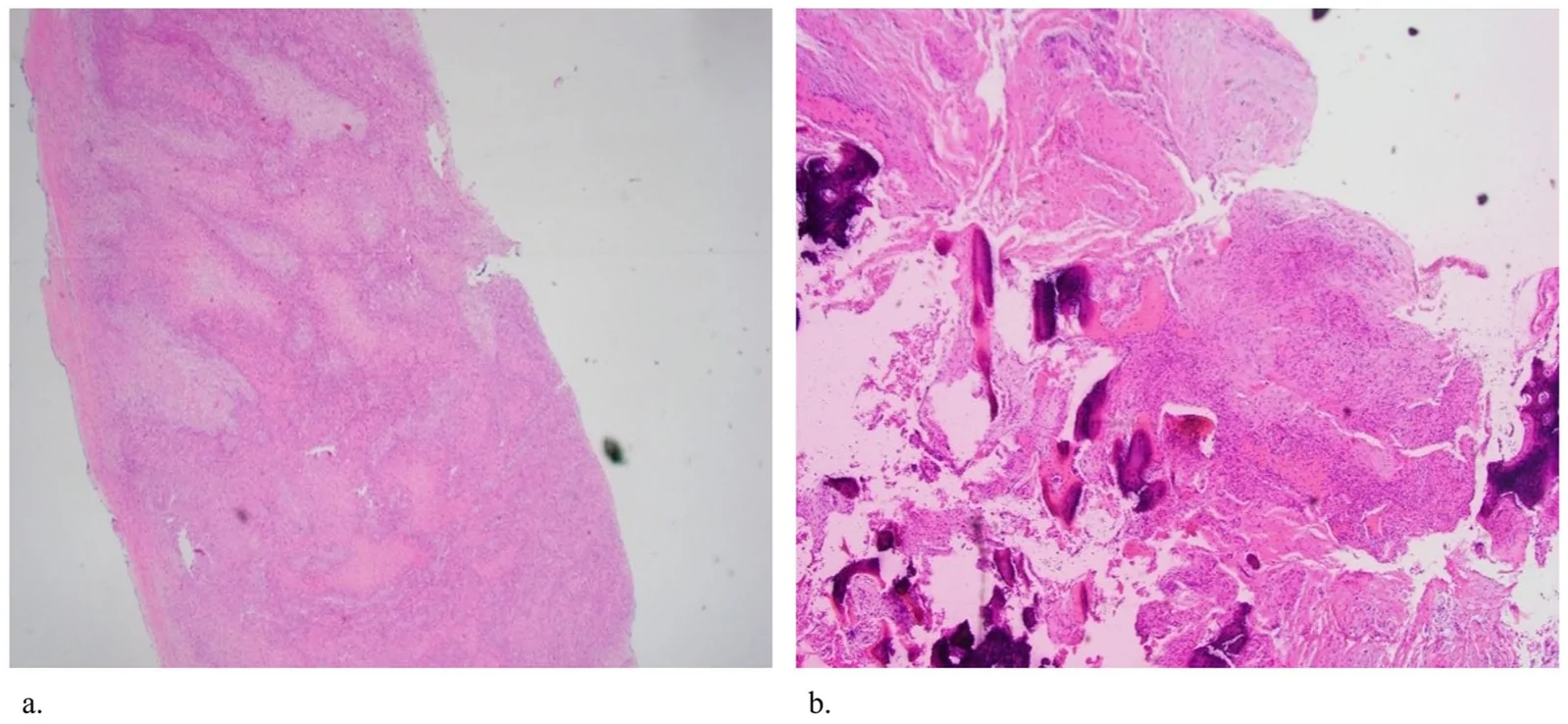
(A) Hypercellular, myxoid stroma dominated by spindle-shaped fibroblast proliferation. (B) Irregular, mineralised bony trabeculae and pink osteoid islands rimmed by active osteoblasts, with a lack of zoning pattern.

At review 6 days later, the wound was healing well; however, digital range of motion was reduced, and fingertip sensation altered (Figure 3). Repeat histology of the initial biopsy specimen confirmed irregular osteoid trabeculae with osteoblastic rimming and associated fibroblastic proliferation demonstrating nodular fasciitis–like changes, with immunopositivity for SMA (Figure 4). A preliminary diagnosis of FOPD was made.

**Figure 5.**
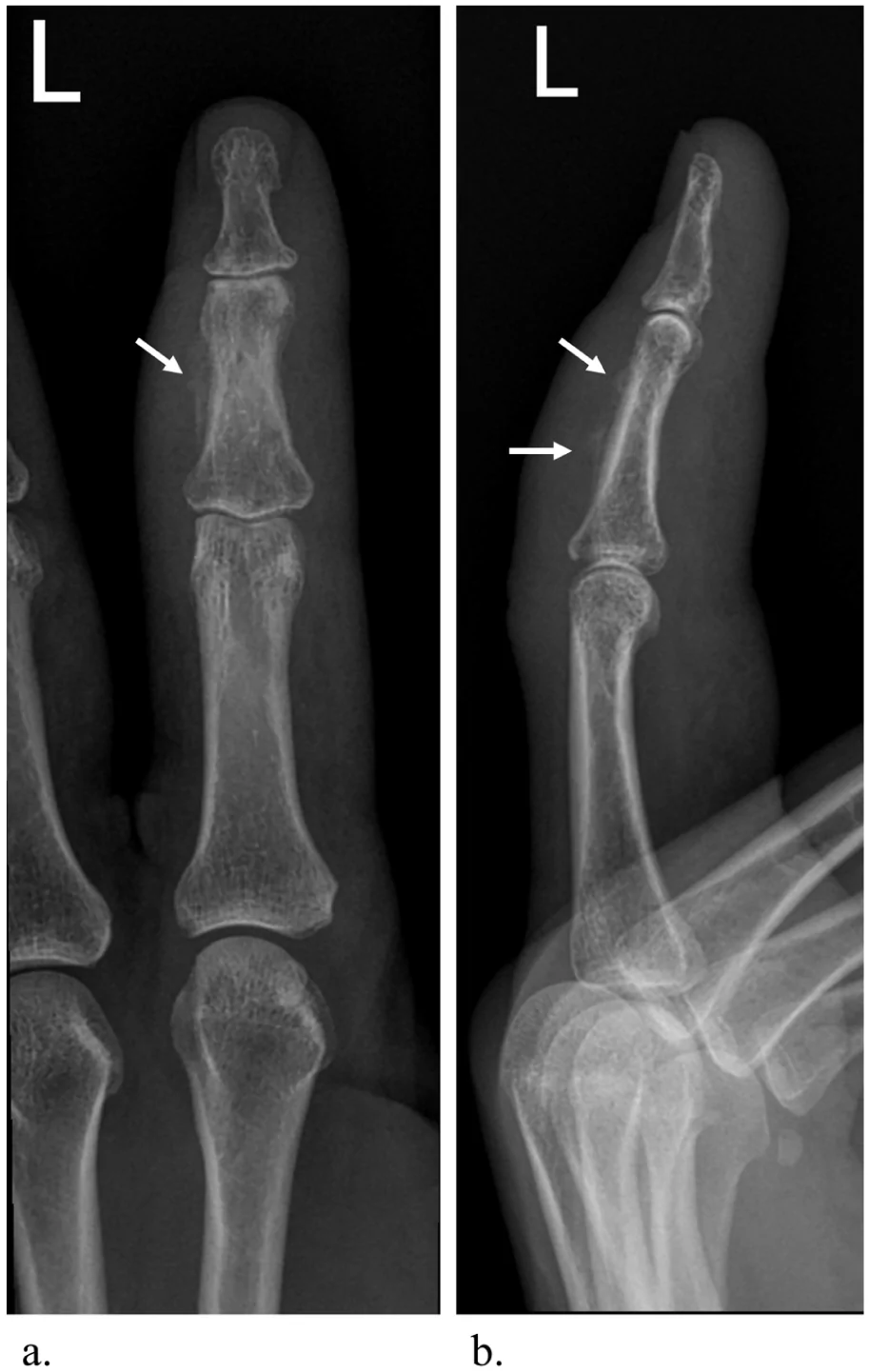
Left index finger radiographs ((A) posteroanterior and (B) lateral) 4 weeks post initial excision of fibro-osseous pseudotumour of the digit, with recurrent soft-tissue swelling. Focal intralesional/periosteal mineralisation (white arrows) tracking along the dorsal/ulnar cortex of the mid to distal shaft/neck of the second middle phalanx with significant overlying soft-tissue swelling. No erosion of the cortex or abnormality interphalangeal joints on radiography.

**Figure 6.**
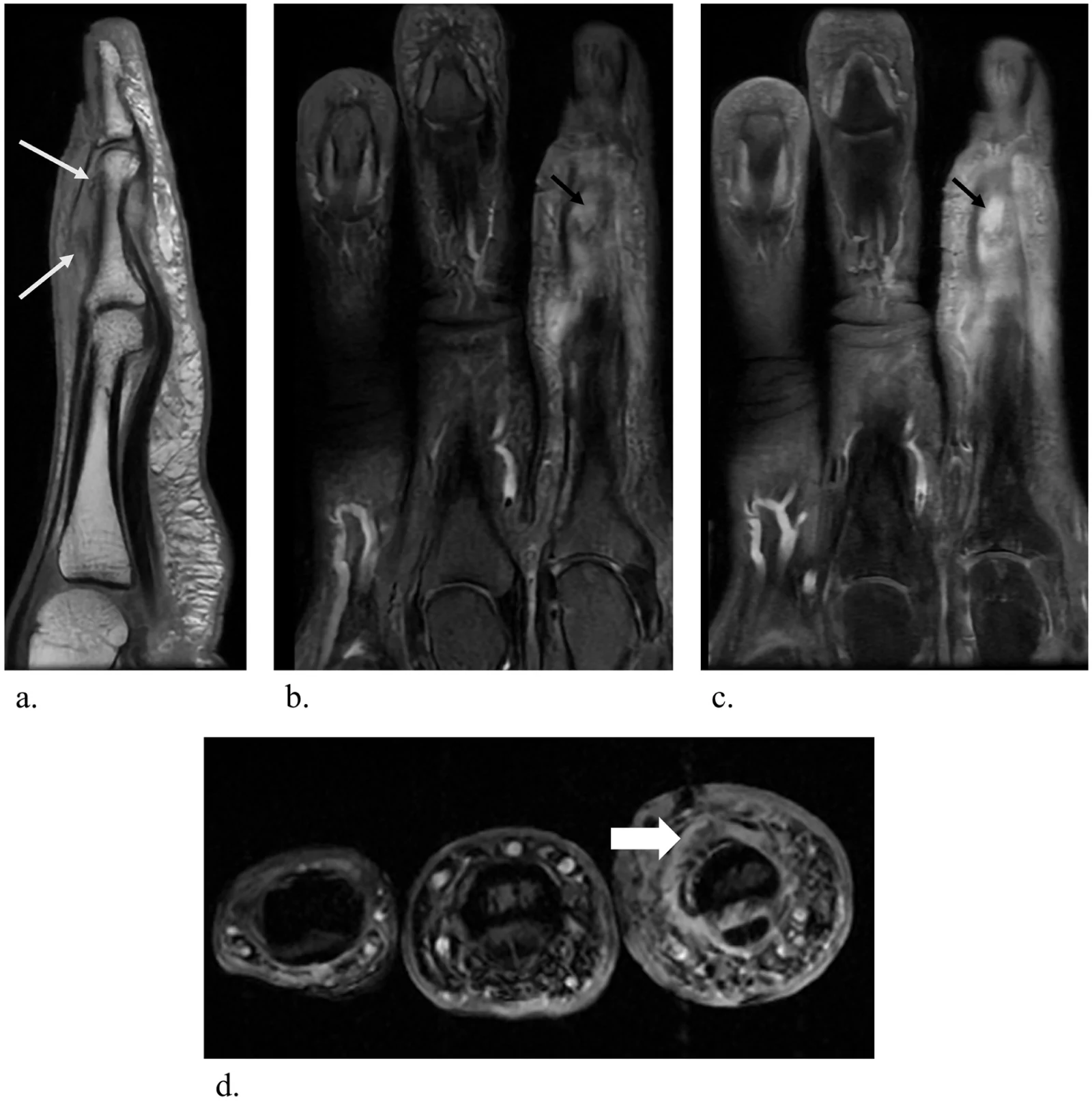
Magnetic resonance imaging performed 4 weeks post initial excision: additional sequences, including T2* and diffusion-weighted imaging, were performed due to suspicion of recurrence of fibro-osseous pseudotumour of the digit (FOPD). (A) Sagittal T1, (B) coronal T2 FS, (C) coronal T1 FS post-gadolinium, and (D) axial T2*. Nodular focal T2 hyperintense/enhancing solid lesion (black arrows) overlies the dorsal aspect of the shaft/neck of the second middle phalanx, compatible with FOPD recurrence. Sagittal T1 (A) and axial T2* (D) sequences confirm low T1 signal from mineralisation (thin white arrows) and susceptibility artefact (thick white arrow) overlying the dorsal cortex.

Over the next 2 weeks, the patient reported fluctuating swelling and pain. Plain radiographs revealed cortical thickening and periosteal reaction without cortical erosion or medullary involvement (Figure 5). Magnetic resonance imaging demonstrated cortical and subperiosteal enhancement with a nodular DIP lesion on the background of diffuse periosteal mineralisation (Figure 6). These findings were consistent with recurrent FOPD, although tenosynovitis or flexor space infection could not be excluded.

At 6 weeks post the index operation, the patient underwent repeat surgery with complete excision of the lesion down to normal bone and irrigation of the flexor sheath. Histopathology of a biopsy from the repeat surgery confirmed FOPD, with no evidence of malignancy. Post-operatively, the patient developed a 10° DIP joint extension lag, proximal interphalangeal joint stiffness, and decreased sensation along the dorsoulnar aspect of the affected finger. There were no signs of infection. The patient gradually obtained functional improvement with hand therapy and opted against additional surgery. At 4 months, he reported a Disability of the Arm, Shoulder, and Hand score of 11, with normal grip strength.

## Discussion

Fibro-osseous pseudotumours of the digit are rare and diagnostically challenging lesions. Their clinical and radiological resemblance to infection and malignancy frequently leads to initial misdiagnosis, as demonstrated in the reported case. This pattern was consistent across the literature, with many cases first mistaken for infection,^1,5-7^ granulomatous processes,^12,17^ or extraskeletal osteosarcoma.^1,3,5-7,9,10,14-27^ Such diagnostic uncertainty risks delay in definitive treatment and, more seriously, unnecessary radical intervention, such as amputation.

This review consolidates findings from 134 reported cases, highlighting consistent clinical themes. Fibro-osseous pseudotumour of the digit typically affects young to middle-aged adults, with a slight female predominance, and presents as a rapidly enlarging, painful digital mass. Swelling, erythema, and tenderness are common, while ulceration is infrequent. A history of antecedent trauma or repetitive use was noted in some cases, but this association remains inconsistent and insufficient to establish causality.

Histopathology remains the cornerstone of diagnosis. The characteristic triad of fibroblastic proliferation, peripheral immature osteoid formation, and variable cytological atypia was reported in most cases.^3,5-7,9,10,14-18,20,23-26,28^ Absence of a zonal pattern helps distinguish FOPD from myositis ossificans,^1,3,4,6,7,9,12,13,24,27^ while the lack of continuity with underlying bone separates it from subungual exostosis.^1,5-7,17^ Immunohistochemistry, though not widely reported, supports a myofibroblastic phenotype with SMA and calponin positivity. More recently, the identification of USP6 gene rearrangements has emerged as a potentially valuable adjunct.^11,24,27,28^ Although not universally present, USP6 fusions were detected in most tested cases, supporting their role as a diagnostic marker and raising the possibility of future applications as a prognostic indicator.

Radiological findings reinforce the benign but deceptive nature of FOPD. Plain radiographs and computed tomography scans often show well-circumscribed soft-tissue masses with periosteal reaction, while MRI typically demonstrates T1 hypointensity and T2 hyperintensity. These features, although non-specific, assist in excluding aggressive osseous lesions. Notably, lesions rarely invade cortical bone, further helping distinguish them from sarcomas.

Surgical excision remains the gold standard of treatment and is usually curative. Recurrence is uncommon but has been reported, almost exclusively following incomplete excision.^1,5-7,13,18,19,21^ In our case, recurrence within weeks of initial debridement and sampling emphasised the importance of definitive diagnosis and complete resection. Functional outcomes across the literature were generally favourable, though few studies provided standardised reporting. Our case adds detail by demonstrating near-complete recovery of grip strength and function but with persistent sensory deficit and extension lag, underscoring the value of long-term outcome reporting.

This review highlights several important implications for clinical practice. First, early biopsy should be considered in rapidly growing digital lesions with atypical features to minimise the risk of misdiagnosis and overtreatment. Second, awareness of FOPD among clinicians is essential to avoid unnecessary radical surgery and incorrect antibiotic treatment. Finally, the integration of histopathology with emerging molecular diagnostics, such as USP6 analysis, may improve diagnostic certainty in ambiguous cases.

The study has limitations. Despite pooling 134 cases, the available literature consists almost exclusively of case reports and small series, limiting the strength of generalisations. Reporting of long-term outcomes, functional recovery, and recurrence rates was inconsistent, and molecular testing was not universally available. Nevertheless, this synthesis provides the most up-to-date overview of FOPD.

In summary, FOPD should be considered in the differential diagnosis of rapidly enlarging digital lesions. Accurate diagnosis relies on a combination of clinical suspicion, imaging, histopathology, and, where available, molecular testing. Complete excision remains the cornerstone of management, with recurrence primarily linked to incomplete resection. Future research should focus on clarifying the pathogenesis of FOPD, standardising diagnostic criteria, and establishing evidence-based guidelines for surgical management and follow-up.
